# The association between structure-function relationships and cognitive impairment in elderly glaucoma patients

**DOI:** 10.1038/s41598-017-07714-7

**Published:** 2017-08-02

**Authors:** Megumi Honjo, Jiro Numaga, Tadashi Hara, Ryo Asaoka

**Affiliations:** 1 Department of Ophthalmology, the University of Tokyo Graduate School of medicine, Tokyo, Japan; 2 Department of Ophthalmology, Tokyo Metropolitan Geriatric Hospital, Tokyo, Japan; 3 Hara Eye Clinic, Tochigi, Japan

## Abstract

Accurate measurement of visual field (VF) is important in accessing glaucoma, however this may not be achieved in patients with dementia or mild cognitive impairment (CI). We investigated the association between CI and structure-function relationships in elderly glaucoma patients. The study included 94 eyes of 51 glaucoma patients aged ≥75 years with no diagnoses of dementia. CI was assessed using the Mini Mental State Examination (MMSE). Using the leave-one-out cross-validation, the mean deviation (MD) of the Humphrey 30-2 VF was predicted from measurements of optical coherence tomography, and the relationship between the squared prediction error and the MMSE score, together with age, fixation loss (FL), false positive (FP), and false negative (FN) percentages that were analyzed using the linear mixed model. A high prevalence of MCI or dementia was observed in the elderly population. The squared prediction error value of the MD was 17.0 ± 21.1 (mean ± standard deviation). The squared prediction error increased with decreasing MMSE total score, but age, FL, FP, and FN were not related. Careful consideration is needed when interpreting the VF results of these patients, because VF can be over- or underestimated, as suggested by the decreased structure-function relationships.

## Introduction

Glaucoma is the second leading cause of blindness in the world^1^. It is characterized by progressive visual field (VF) damage, which affects the quality of life^2^. Glaucoma was present in 60.5 million people worldwide in 2010, but this number is estimated to rise to 79.6 million by 2020, because of the aging population and because the prevalence of ocular diseases increases with increasing age^3–6^. The aged population faces other problems, such as cognitive impairment and dementia, which affect their independent and active lives, and may eventually be fatal^7^. Dementia is a decline of cognitive function severe enough to interfere with social function, and is not associated with changes in consciousness. Alzheimer’s disease (AD) is the most common form of dementia, and the prevalence of dementia has been reported to increase rapidly with advancing age^8^. Dementia reportedly affects 36 million people globally^9^. The true prevalence of missed and delayed diagnoses of dementia is unknown, but is likely high^10^. Additionally, mild cognitive impairment (MCI), which is a risk factor for the development of dementia but not sufficient for a diagnosis of dementia^11^, has become a major health problem among older patients because of its high prevalence in the aged population. Approximately 15–42% of patients aged ≥65 years are estimated to have MCI, and approximately 5–15% of these patients progress to dementia annually^12^, suggesting that high rates of diagnosed/undiagnosed dementia or MCI occur in glaucoma patients.

Reliable VF results are required to achieve an accurate diagnosis of glaucoma as early as possible^13, 14^. However, this may not be achieved in patients with dementia or MCI. Fixation losses (FLs), false positives (FPs), and false negatives (FNs) can be reliably measured using the Humphrey Field Analyzer (HFA; Carl Zeiss Meditec, Dublin, CA, USA). These indices are important clinical markers. Elevated FLs can mask early scotoma^15, 16^, increased FP errors indicate ‘trigger-happy’ patients, and high FNs may suggest patient inattention or fatigue during the VF examination^17–19^. Previous studies have suggested the usefulness of these indices^20, 21^, but more recent studies have reported their limitations in reliably estimating the VF^16, 22–24^.

Recent developments in optical coherence tomography (OCT) have enabled evaluation of the circumpapillary retinal nerve fiber layer (cpRNFL) and the macular ganglion cell complex (GCC) thicknesses^25–33^. Previous studies have reported that these structural measurements are related to the VF^28, 29, 34–41^. However, these structure-function relationships can be affected by other factors. For example, we previously reported that eye movement, as measured by gaze tracking using the HFA, resulted in improved characterization of structure-function relationships^42^. We recently reported significant correlations between structure-function relationships in elderly glaucoma patients when reliable perimetry was performed^43^. However, VF examinations are often unreliable in elderly patients^44^, and cognitive status in elderly patients could be related to the reliability of the VF measurements.

The Mini-Mental State Examination (MMSE) is a widely used cognitive test that is useful for examining patients with an increased risk of dementia^45^. In the present prospective study, cognitive impairment was assessed using the MMSE in elderly glaucoma patients who did not have a diagnosis of dementia. The objectives of this study were to confirm the prevalence of cognitive impairment in elderly glaucoma patients, to identify possible relationships between cognitive decline and reliable measurements of the VF in these patients, and to determine whether cognitive impairment was related to structure-function relationships in the same patients.

## Methods

### Subjects

This prospective, cross-sectional study was conducted at the Tokyo Metropolitan Geriatric Hospital and the Hara Eye Clinic. Only patients ≥75 years of age with no history of dementia were recruited. The Institutional Review Board and Ethics Committee of the institute approved the study, and the study protocol adhered to the tenets of the Declaration of Helsinki. Written informed consent for both study participation and publication of ocular examination images was obtained from each participating patient.

The study initially included 104 eyes of 54 OAG glaucoma patients. All patients visited the glaucoma clinic from July 2015 to November 2015. They fulfilled the following criteria: (1) glaucoma was the only disease causing VF damage; (2) axial length was between 26.0 mm and 21.0 mm; (3) patients underwent at least three VF tests, prior to the current study (to mitigate learning effects); and (4) logMAR visual acuity ≤0.5^46^. All VFs were measured using the HFA (30-2 Swedish Interactive Threshold Algorithm, standard program) and those with unreliable VFs defined as FL > 33%, FP > 33%, or FN > 33% were excluded^47^. Patients with successful cataract surgery and patients with clinically insignificant cataract were included in the study. After providing written informed consent, all patients underwent an ocular examination including an autorefractometry examination, measurement of best-corrected visual acuity, a slit-lamp examination, measurement of the axial length using the IOL Master (Carl Zeiss Meditec), measurement of the intraocular pressure, dilated fundoscopy, VF measurement, MMSE, and a spectral domain (SD)-OCT examination using a RS-3000 Advance OCT, software version 1.4.2.1 (NIDEK, Gamagori, Japan). All examinations were conducted within 1 month of the VF measurements.

### Assessment of cognitive function

Cognitive function was assessed using the MMSE, which was developed by Folstein *et al*. in 1975 and is widely used as a brief screening test for dementia and as a measure of global cognitive function^48^. It focuses on five downstream items: orientation, memory, concentration, language, and design capacity. It includes 11 questions, and was performed by an experienced examiner^49^.

The MMSE total score ranged from 0–30, with lower scores indicating poorer cognitive ability. Patients with a score of ≤23 points were classified as having cognitive impairment^50, 51^. Using the MMSE total score, any score ≥28 points indicated normal cognition, and scores <28 points indicated MCI (24–27 points) with moderate (10–23 points) or severe (≤9 points) cognitive impairment. A score of ≤9 points was considered to be almost diagnostic of dementia^50–52^.

### OCT measurements

OCT examinations were performed using an RS-3000 Advance SD-OCT as previously described^53, 54^. For cpRNFL imaging, raster scanning over a 6 × 6 mm^2^ area centered on the optic disc center was performed at a scan density of 512 A-scans (horizontal) × 128 B-scans (vertical). For wide area three-dimensional imaging of the macula, raster scanning was performed over a 30° × 30° area (equivalent to a 9 × 9 mm^2^ area in the Gullstrand model eye). The cpRNFL/GCC thickness was measured using the built-in software. An experienced examiner (MH) confirmed the validity of the image segmentation, and any images with motion artifacts or incorrect segmentation were excluded from analyses. Only images with a signal strength index >50 were included in analyses.

### Statistical analysis

**Figure 1:**
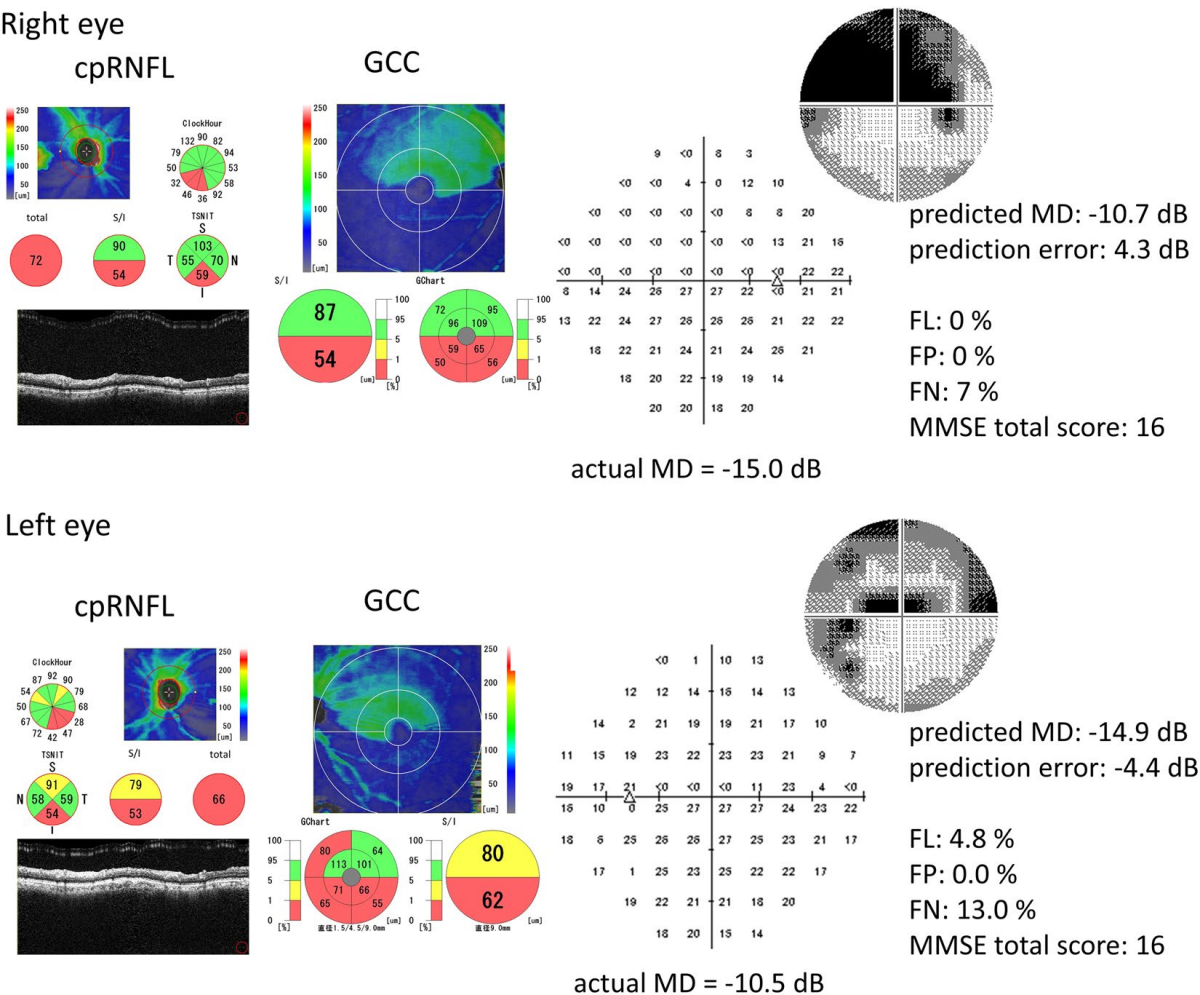
A case example (86 year old male). MD values were predicted from OCT determined cpRNFL thickness and average GCC thickness through the leave-one-out cross-validation; the predicted MD value was calculated from the remaining 50 patients (92 eyes). Despite the reliable FL, FP and FN results, a markedly over- (right eye) and under- (left eye) prediction was obtained. This patient had the lowest MMSE total score (16). MD: mean deviation, OCT: optical coherence tomography, cpRNFL: circumpapillary retinal nerve fiber layer, GCC: ganglion cell complex, FL: fixation loss, FP: false positive, FN: false negative, MMSE: Mini Mental State Examination.

To determine how MMSE total score, FL, FP, and FN affected the degree of structure-function relationships, a linear mixed model was constructed between the average cpRNFL thickness and the average GCC thickness against the mean deviation (MD). A linear mixed model was applied to a nested dataset in the current study, and patients were treated as a ‘random effect’ because both eyes were included in analyses. Then, the prediction error was calculated using the leave-one-out cross-validation method, whereby the data from one or both eyes of a single patient were used for validation, and the data from the remaining subjects were used for training. This procedure was then repeated until each OAG patient in the original sample was used once as validation data. Thus, for each individual, only the data from all other subjects were used in the prediction. Finally, using the variables of age, FL, FP, FN, and MMSE total score, the optimal linear mixed model for the raw prediction error value, the squared prediction error, and the prediction error value (see Fig. 1) were selected among 2^5^ patterns (optimal model_prediction error_, optimal model_squared prediction error_), using the second-order bias-corrected Akaike information criterion index which is a correction for finite sample sizes^55^. All statistical analyses were performed using the statistical programming language ‘R’ version 3.2.3 (Foundation for Statistical Computing, Vienna, Austria).

### Data Availability

All data generated or analysed during this study are included in this published article.

## Results

**Figure 2:**
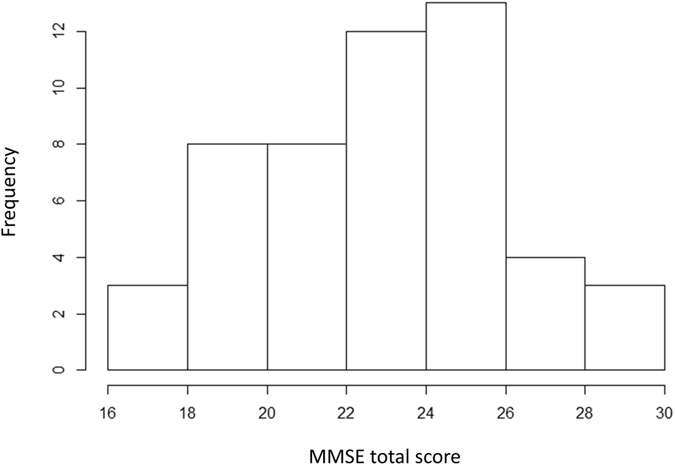
Histogram of Mini Mental State Examination (MMSE) total scores. The MMSE total score was 23.3 ± 3.2 (range, 16.0–29.0) (mean ± standard deviation).

Among the 104 eyes of 54 patients, 94 eyes of 51 patients had FL < 33%, FP < 33% and FN < 33%. Table 1 lists the demographic data of patients: 58 eyes were phakic and 36 eyes were pseudophakic. Figure 2 shows a histogram of the MMSE total scores. Among the 51 patients, five patients (9%) scored 28–30 points (normal); 22 patients (41%) scored 24–27 points (MCI), and 24 patients (44%) scored ≤23 points (severe to moderate CI or almost diagnostic of dementia). No patients scored <15, so there was no severe CI among the patients in the current study.

**Table 1:** Patient demographics.

variables	number
Gender (male:female)	24:27
Age (years), mean ± SD [range]	80.8 ± 3.8 [76 to 90]
Eye (right:left)	47:47
MD (dB), mean ± SD [range]	−6.5 ± 4.7 [−17.3 to 0.9]
cpRNFL thickness (μm) mean ± sd [range]	82.5 ± 14.2 [50.0 to 113.0]
GCC thickness (μm) mean ± sd [range]	77.7 ± 12.0 [50.5 to 103.0]
FL (%) mean ± SD [range]	9.7 ± 9.4 [0.0 to 31.6]
FP (%) mean ± SD [range]	2.1 ± 3.1 [0.0 to 20.0]
FN (%) mean ± SD [range]	5.3 ± 6.5 [0.0 to 20.0]
total MMSE score, mean ± SD [range]	23.3 ± 3.2 [16.0 to 29.0]

SD: standard deviation, MD: mean deviation, cpRNFL: circumpapillary retinal nerve fiber layer, GCC: ganglion cell complex, FL: fixation loss, FP: false positive, FN: false negative, MMSE: Mini Mental State Examination.

**Figure 3:**
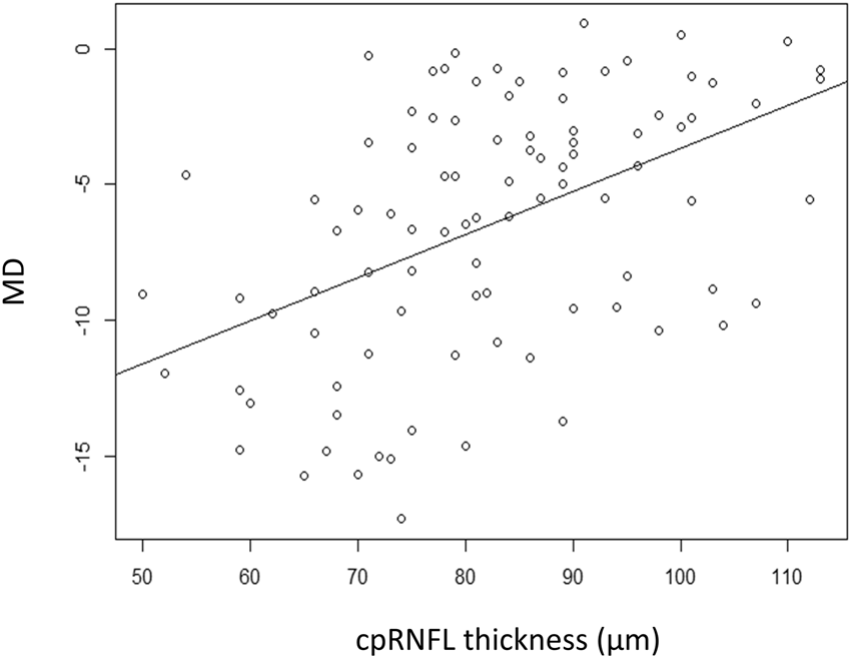
Relationship between the mean deviation (MD) and the circumpapillary retinal nerve fiber layer (cpRNFL) thickness. A significant relationship was observed between the MD and the cpRNFL thickness [MD = −19.6 + 0.16 (standard error, 0.031; p < 0.0001) × cpRNFL thickness (linear mixed model)].

**Figure 4:**
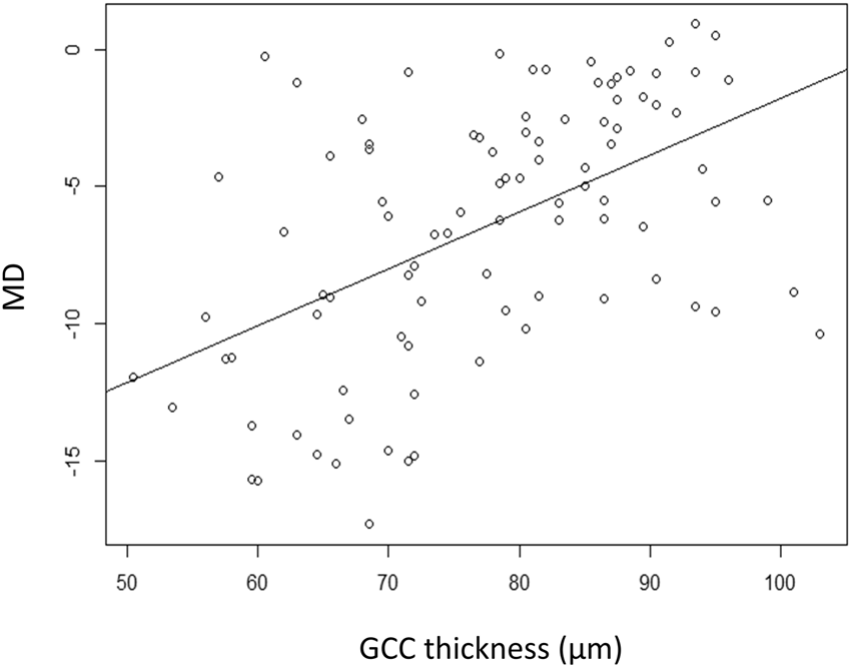
Relationship between the mean deviation (MD) and the ganglion cell complex (GCC) thickness. A significant relationship was observed between the MD and GCC thickness [MD = −22.5 + 0.21 (standard error, 0.036; p < 0.0001) × GCC thickness (linear mixed model)].

**Figure 5:**
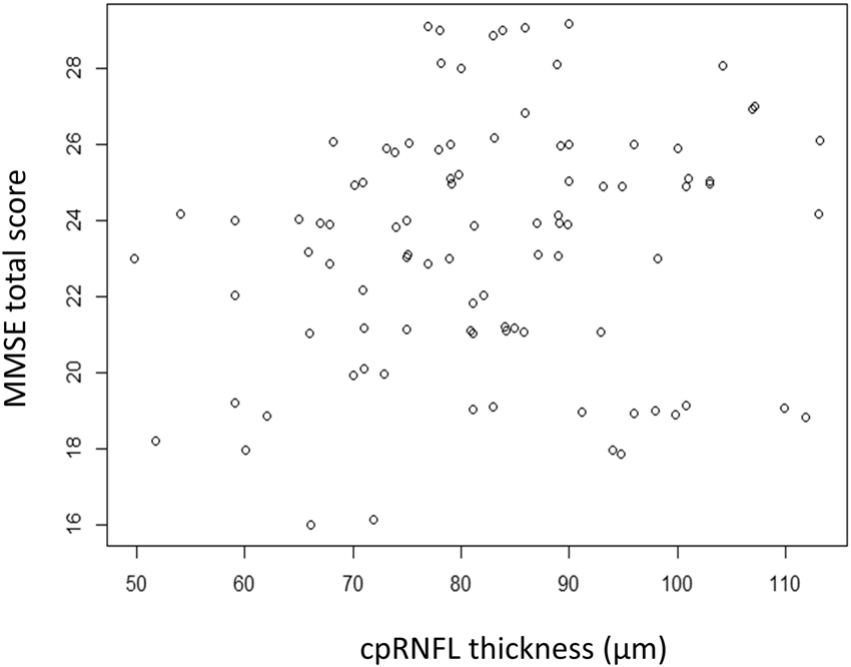
The relationship between the Mini Mental State Examination total score and the circumpapillary retinal nerve fiber layer thickness. No significant relationship was observed (p = 0.17, linear mixed model).

**Figure 6:**
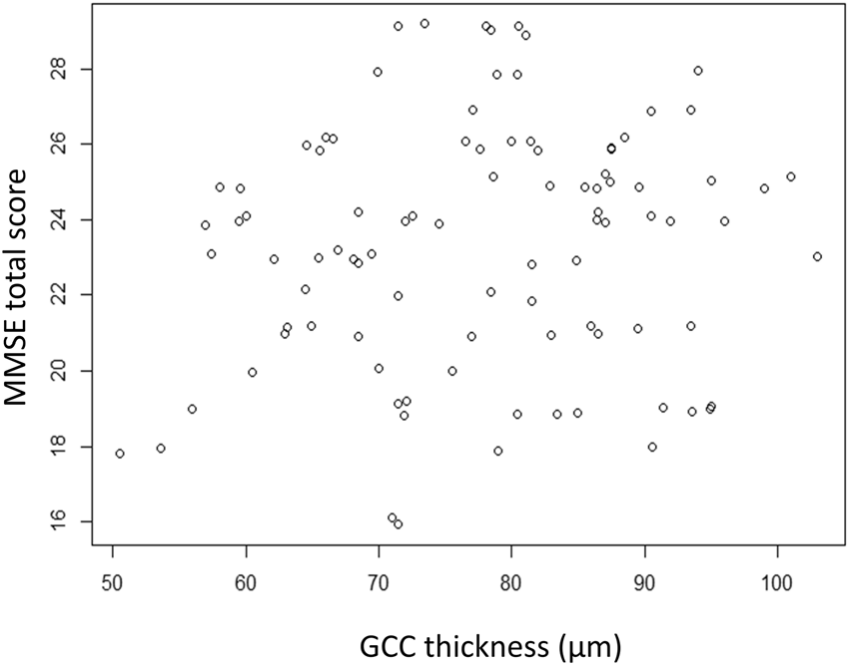
The relationship between the mean deviation and the ganglion cell complex thickness. No significant relationship was observed (p = 0.23, linear mixed model).

Figure 3 shows the relationship between cpRNFL thickness and the MD, which was significant (p < 0.001, linear mixed model). Similarly, a significant relationship was observed between GCC thickness and the MD (p < 0.001; Fig. 4). The MMSE total score was significantly related to age (p < 0.001), but not to MD, FL, FP, or FN (Table 2); this analysis was carried out in 104 eyes of 54 patients including those with FL > 33%, FP > 33% or FN > 33%. As shown in Figs 5 and 6, the cpRNFL thickness and the GCC thickness did not significantly correlated with MMSE total score (p = 0.17 and 0.23, respectively, linear mixed model).

**Table 2:** The relationship between MMSE total score and age, MD, FL, FP, and FN.

dependent variables	independent variable	coefficient	SE	p value
MMSE total score	Age (years)	0.13	0.031	<0.001
MD (dB)	MMSE total score	0.21	0.17	0.22
FL (%)	MMSE total score	−0.059	0.35	0.87
FP (%)	MMSE total score	0.039	0.12	0.75
FN (%)	MMSE total score	−0.31	0.24	0.21

MMSE: Mini Mental State Examination, SD: standard deviation, MD: mean deviation, FL: fixation loss, FP: false positive, FN: false negative.

The multivariable linear mixed model between the MD and the cpRNFL thickness, GCC thickness, and age resulted in the following formula: MD = −26.3 + 0.064 × cpRNFL thickness + 0.15 × average GCC thickness + 0.034 × age. Using the leave-one-out cross-validation, the MD was predicted using cpRNFL thickness, GCC thickness, and age, and the raw prediction error was 0.058 ± 4.2 (−9.7 to 9.9) dB. The optimal model_prediction error_ was constructed by selecting optimal parameters from age, FL, FP, FN, and MMSE total score. MMSE total score, FL, FP, and FN were not selected, and only the intercept existed in the optimal model_prediction error_.

**Figure 7:**
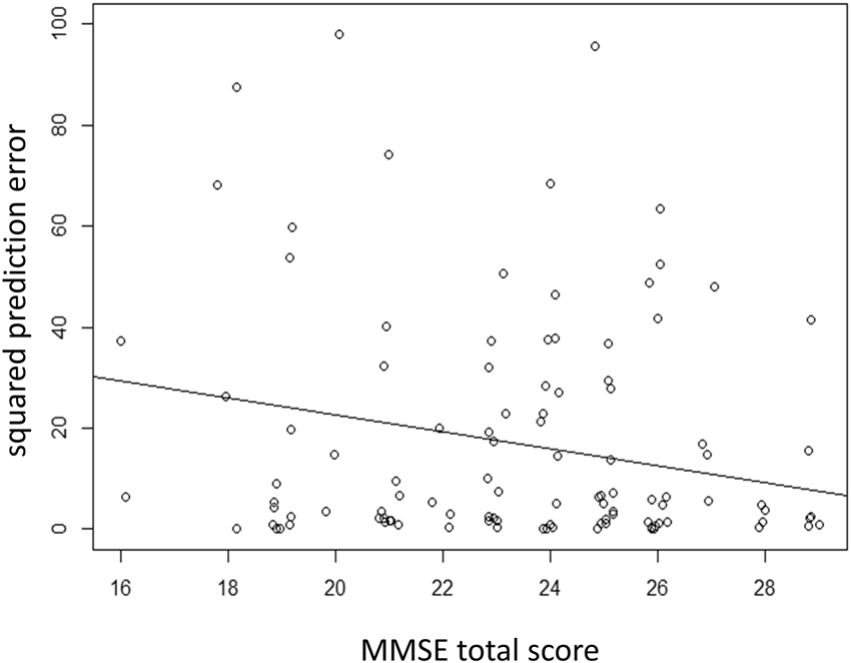
The relationship between the squared prediction error and the Mini Mental State Examination (MMSE) total score. Only the MMSE total score was selected in the optimal model_squared prediction error_. The squared prediction error = 56.4 - 1.7 (standard error = 0.66, p = 0.0012) × MMSE total score.

The squared prediction error was calculated using the leave-one-out cross-validation method [17.0 ± 21.1 (0.0095 to 97.0)]. The optimal model_squared prediction error_ was constructed by selecting optimal parameters using age, FL, FP, FN, and MMSE total score. Only the MMSE total score was selected in the optimal model_squared prediction error_, with the squared prediction error = 56.4–1.7 (standard error = 0.66, p = 0.0012) × MMSE total score (AICc = 840.5). The relationship between squared prediction error and the MMSE total score is shown in Fig. 7. The optimal model_squared prediction error_ was significantly better than the model only with intercept (no independent variables included: AICc = 844.8): p = 0.011 (ANOVA test)^56^. None of the models between squared prediction error and FL, FP, FN and age were significantly better than the model only with intercept (p values between 0.35 and 0.95, ANOVA test), and adding any combinations of the four variables resulted in the decrease of AICc (AICc values were between 846.2 and 847.0).

## Discussion

The present study measured the cognitive impairment in 94 eyes of 51 OAG patients >75 years of age, together with OCT and VF measurements. The cognitive impairment in elderly glaucoma patients and the relationship between cognitive decline and reliability measurements of the VF were assessed. The MD of the VF was predicted using OCT-measured cpRNFL and GCC thicknesses using the leave-one-out cross-validation method, and the relationship between the prediction error and the MMSE score was determined. The results revealed that the squared prediction error increased with decreasing MMSE score, indicating that prediction of the MD from OCT parameters was less accurate in these patients.

The onset of AD or dementia is sometimes difficult to diagnose, because it can be difficult to detect in its early stages. Furthermore, there is no effective treatment once the disease becomes clinically evident^57^. In a population-based study, 15–42% of the patients ≥65 years of age were estimated to have MCI^12^, and the prevalence of cognitively impaired individuals ≥65 years of age was estimated at 15% in Japan^58^. The results of the present study suggest that the prevalence of undiagnosed dementia or MCI is relatively high among elderly glaucoma patients. No patients were diagnosed with severe CI in the current study. However, of the 51 patients, only five (9%) had a normal MMSE score; 22 (41%) were classified as MCI, and 24 (44%) were classified as having moderate CI or suspected dementia (Fig. 2), although MMSE scores did not decrease with increased age >75 years (Table 2). It is important to note that none of the patients in the current study had been diagnosed with dementia; they lived their daily lives without any special assistance. Several previous studies have reported visual impairment-related cognitive hypofunction based on longitudinal research^59, 60^. However, one recent study found no significant association between visual impairment and cognitive function in a 10-year follow-up study^61^. Further studies will therefore be needed to determine the association between glaucomatous visual impairment and cognitive function. In the Japanese governmental medical insurance system, individuals ≥75 years of age are classified as ‘latter-stage elderly,’ so increased attention should be addressed to these patients.

We have previously reported that the FL and FP were related to overestimation of the VF^42^; however this relationship was not observed in the current study. This may suggest that the usefulness of these reliability indices is limited in a population ≥75 years of age. In contrast, the squared prediction error was related to MMSE total score (Fig. 7), and an increased squared prediction error was found among patients with low MMSE total scores (the optimal model_squared prediction error_). This result suggests that patients who cannot obtain high MMSE total scores, even if they have not been diagnosed with dementia or categorized as MCI, tend to perform inaccurately with overly high or overly low VF measurements, suggesting that careful clinical consideration is needed when interpreting the VF results of patient populations with a high prevalence of MCI and early to moderate dementia. MMSE total score were selected as significant parameters in the model_squared prediction error_, however not in the optimal model_prediction error_. This suggests VFs are over-/under-estimated probabilistically in those with low MMSE score. In a future study, the effects of MMSE total scores on the test-retest reproducibility of VF tests should be examined. As squaring the prediction error to calculate the squared prediction error changes the distribution and the value may not follow normal distribution. We used the linear mixed model which requires normal distribution, we log-transformed the squared prediction error. As a result, only the MMSE total score was included in the optimal model again (data not shown in Result).

We recently reported that it is useful to include gaze tracking data, in addition to the FL, FP, and FN, to investigate test-retest reproducibility^61^ and over-and underestimation of the VF^62, 63^. Recent studies have suggested that the FL, FP, and FN have limited usefulness in estimating the reliability of the VF^16, 20–22^. Consistent with this possibility, in this study we observed no significant relationship between MMSE total score and any of the FL, FP, and FN values. Further studies should be performed to determine the usefulness of gaze tracking in the elderly population in assessing the reliability of the VF results, and the influence of cognitive impairment on gaze tracking.

In elderly patients, the VF with a narrower testing area, such as the 10-2 HFA VF, may be more accurately measured, because of various ocular conditions, including ptosis and miosis of the pupil. Also, the GCC scanning area corresponds mainly to the 10-2 VF. We recently reported that cpRNFL and GCC thicknesses were significantly correlated with HFA VF sensitivity in elderly glaucoma patients when reliable VF measurements were obtained^43^. GCC thicknesses especially were strongly correlated with sensitivities in all test points within the central 5.8° measured with the HFA 10-2 VF.

This study did not include use of the 10-2 HFA VF, which is a limitation that should be further investigated in future studies. Additionally, the MMSE consisted of 11 questions measures that test five areas of cognitive function: orientation, registration, attention and calculation, recall, and lauguage, but we did not investigate each of the scores, because of the small number of items (between 1–6). Several previous studies have reported that the thickness of the cpRNFL or GCC in subjects with AD and MCI is significantly thinner than in normal controls, so OCT could be a potentially useful diagnostic tool in the evaluation and follow-up of AD patients^64, 65^. In the present study, there was no significant relationship between MMSE total score and the thicknesses of cpRNFL and GCC (Figs 4 and 5). The results of the present study therefore do not suggest that retinal nerve fiber degeneration caused by both glaucoma and central nervous system degeneration occur concurrently with thinning of the RNFL and GCC thicknesses measured by OCT. However, further studies with larger sample sizes and precise evaluations of cognitive functions including pathological examinations and neuroimaging will be needed to confirm the existence of a correlation between structural changes and cognitive impairment. Furthermore, care is required to optimize the value of OCT to follow-up elderly glaucoma patients.

In conclusion, a high prevalence of early to moderate cognitive impairment was observed in the elderly study population. Careful consideration is needed when interpreting the VF measurements of these patients, because the VF can be over- or underestimated, as suggested by the inconsistent structure-function relationships.
